# Recovery of Acid and Base from Sodium Sulfate Containing Lithium Carbonate Using Bipolar Membrane Electrodialysis

**DOI:** 10.3390/membranes11020152

**Published:** 2021-02-22

**Authors:** Wenjie Gao, Qinxiang Fang, Haiyang Yan, Xinlai Wei, Ke Wu

**Affiliations:** 1 Collaborative Innovation Center for Environmental Pollution Precaution and Ecological Rehabilitation of Anhui, School of Biology, Food and Environment Engineering, Hefei University, Hefei 230601, China; wenjieg96@163.com (W.G.); saltwaterfang@gmail.com (Q.F.); 2 CAS Key Laboratory of Soft Matter Chemistry, Collaborative Innovation Center of Chemistry for Energy Materials, School of Chemistry and Material Science, University of Science and Technology of China, Hefei 230026, China; oceanyan@ustc.edu.cn

**Keywords:** lithium carbonate, BMED, clean production, current efficiency, recycling

## Abstract

Lithium carbonate is an important chemical raw material that is widely used in many contexts. The preparation of lithium carbonate by acid roasting is limited due to the large amounts of low-value sodium sulfate waste salts that result. In this research, bipolar membrane electrodialysis (BMED) technology was developed to treat waste sodium sulfate containing lithium carbonate for conversion of low-value sodium sulfate into high-value sulfuric acid and sodium hydroxide. Both can be used as raw materials in upstream processes. In order to verify the feasibility of the method, the effects of the feed salt concentration, current density, flow rate, and volume ratio on the desalination performance were determined. The conversion rate of sodium sulfate was close to 100%. The energy consumption obtained under the best experimental conditions was 1.4 kWh·kg^−1^. The purity of the obtained sulfuric acid and sodium hydroxide products reached 98.32% and 98.23%, respectively. Calculated under the best process conditions, the total process cost of BMED was estimated to be USD 0.705 kg^−1^ Na_2_SO_4_, which is considered low and provides an indication of the potential economic and environmental benefits of using applying this technology.

## 1. Introduction

Lithium carbonate, an inorganic compound, exists as colorless monoclinal crystals or a white powder. Lithium carbonate is the basic material required for the production of secondary lithium salt and lithium metal products, so it has become the most-consumed lithium product in the lithium industry. Other lithium products are essentially downstream products of lithium carbonate [1,2]. In particular, the development of new energy vehicles, most of which are powered by lithium batteries, has greatly promoted the use of lithium. Global lithium consumption has increased rapidly from 48,100 tons in 2018 to 57,700 tons in 2019 [3]. The price of battery-grade lithium carbonate has risen from USD 6500 per ton in 2015 to USD 13,000 per ton in 2019 [3]. The increasing demand and high profits have led to a surge in lithium production.

**Scheme 1:**
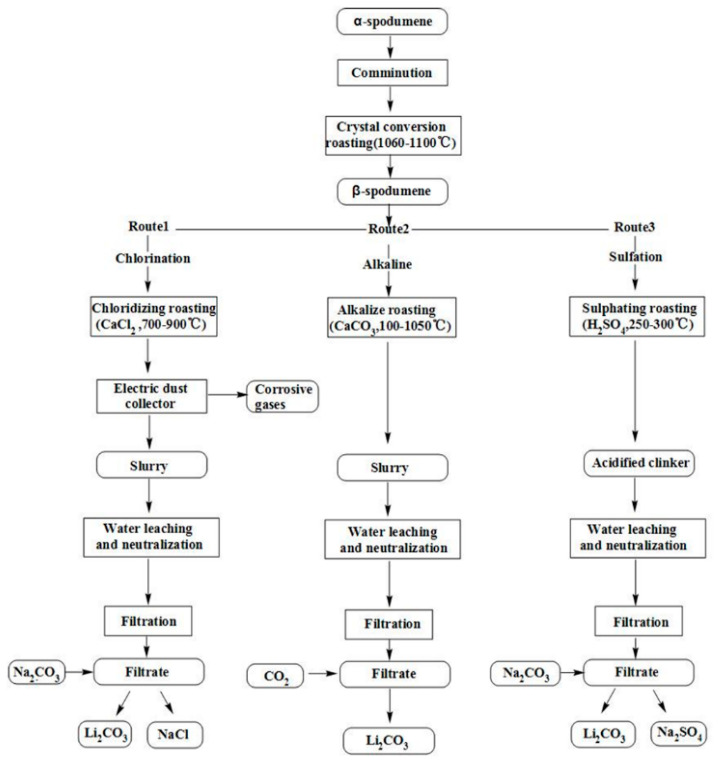
Reaction scheme for the synthesis of lithium carbonate by chlorination roasting, acid roasting, and alkali roasting.

As is well known, the traditional extraction processes of lithium resources from ores mainly comprise chlorination roasting, alkali roasting, and sulfuric roasting methods; heir synthetic routes are shown in Scheme 1 [4,5,6,7,8]. In the first route, the chlorination roasting method, the obtained chlorinated products are difficult to collect, and the furnace gas is highly corrosive. Economic costs are high because of the complexity of the involved processes [4,7]. In the second route, the alkaline roasting method, difficulties in operating and maintaining equipment develop due to the high energy consumption for evaporation, the high requirement for temperature control, the low recovery rate of lithium, and strong flocculability of the sludge after water leaching [6]. The last route, the sulfuric roasting method, is currently the industry standard for the preparation of lithium carbonate, being relatively mature and commonly used. β-Spodumene and sulfuric acid are calcined under conditions of 250–300 °C via the displacement reaction of lithium sulfate from soluble and insoluble gangue. Sodium hydroxide is added to the calcined product to adjust the pH to neutralize excess sulfuric acid and remove impurities [5,8]. Then, pure lithium sulfate is obtained by further removing calcium and magnesium with soda ash. Sodium carbonate is then added to obtain lithium carbonate. Sulfuric roasting has strong adaptability to various raw materials and can handle many kinds of lithium-bearing ores. The process route is simple and easy to operate, and the recovery yield of lithium is very high, reaching more than 90%. However, the route is hampered by subsequent problems. It can be seen that in addition to the massive use of raw materials, the production of sodium sulfate as a byproduct is also a major problem. A large amount of sulfuric acid and NaOH is converted into low-value sodium sulfate. Because of the low purity of the produced waste sodium sulfate, subsequent processing has become a burden that will undoubtedly increase the energy consumption of the process and further increase the running cost. Hence, bipolar membrane electrodialysis (BMED) may be a feasible choice to overcome these challenges [9].

A bipolar membrane is composed of an anion membrane and a cation membrane [10,11,12]. The anion and cation membranes are bonded by an intermediate catalytic layer. When a current is applied to the bipolar membrane, water was split into hydrogen ions and hydroxide ions in the interfacial layer of the bipolar membrane [13,14,15]. Electrodialysis (ED) technology is an electromembrane separation process in which anions and cations migrate to opposite electrodes under the action of a direct current electric field, thereby achieving ion separation, concentration, and purification [16,17,18]. BMED technology is based on traditional electrodialysis, in which H_2_O splits into H^+^ and OH^−^ in the interfacial layer of the bipolar membrane [19,20]. BMED can simultaneously achieve high-salinity wastewater desalination and acid and alkali production without introducing other components [21,22]. The produced acid and alkali can be directly or indirectly reused in industrial applications, enhancing the value of high-salinity wastewater. BMED has been researched in waste salt treatment and acid–base reuse in the production processes of neopentyl glycol, ore mining, and phenyl glycine. [21,23,24].

**Figure 1:**
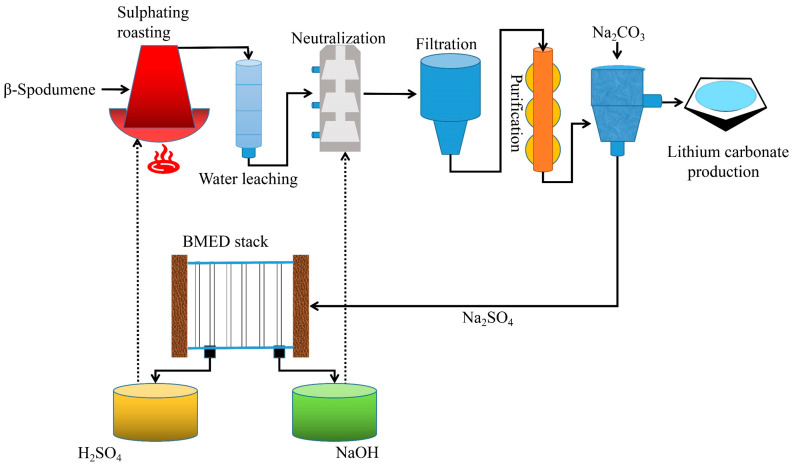
A flow chart of the preparation of lithium carbonate by acid roasting and bipolar membrane electrodialysis (BMED) technology.

In order to solve the problem of the sodium sulfate waste salt produced in the lithium carbonate production process, BMED technology was introduced into the above process to recycle the waste salts, as shown in Figure 1; the waste salt (sodium sulfate) can be converted into sodium hydroxide and sulfuric acid, and both products can be used as raw materials in the upstream process. Therefore, a closed-loop lithium carbonate production process can be achieved by introducing BMED processing. The main purpose of this work is to verify the feasibility of BMED for the treatment of sodium sulfate waste containing lithium carbonate. The influence of feed salt concentration, current density, volume ratio, and flow rate on BMED performance was investigated.

## 2. Experiment 

### 2.1. Materials

We used self-made simulated sodium sulfate waste salt (0.3 mol·L^−1^) containing lithium carbonate; its components are shown in Table 1 [5]. H_2_SO_4_, NaOH, NaCO_3_, and other chemicals used in this study were all analytically pure (Meifeng Chemical Instrument Co. Ltd., Hefei, China). The anion exchange, cation exchange, and bipolar membranes were purchased from Tokuyama Co., Tokyo, Japan. Table 2 lists the main properties of the three membranes used in the experiment.

### 2.2. Setup

**Figure 2:**
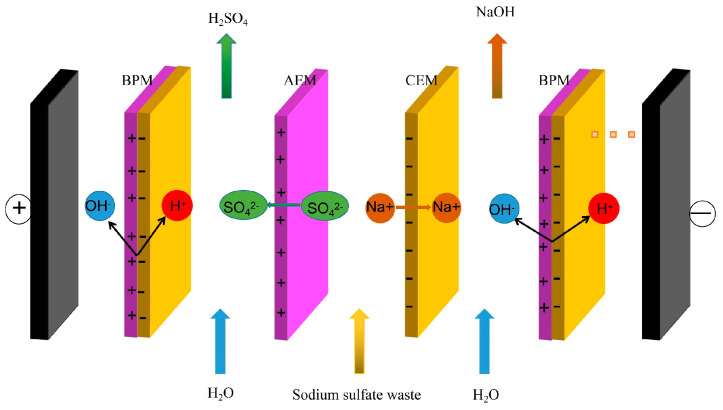
Schematic diagram of the BMED membrane reactor used in the experiment.

A self-assembled BMED stack was used for the experiment. The stack consisted of six bipolar membranes, five anion exchange membranes, and five cation exchange membranes comprising five repeating BM–A–C–BM units, one of which is shown in Figure 2. The effective membrane area of each membrane was 189 cm^2^. The adjacent membranes were separated by a spacer with a thickness of 0.75 mm. A ruthenium-coated titanium electrode plate were connected on either side of the membrane stack, respectively, and the two plates were connected with a DC power supply (WYL1703, Hangzhou Siling Electrical Instrument Co. Ltd., Hangzhou, China). Four circulating loops ensured streams recirculated into the stack and were formed between two neighboring membranes with four peristaltic pumps (Baoding Lead Fluid Technology Co. Ltd., Baoding, China), the salt compartment, acid compartment, base compartment, and electrode compartment. According to different experimental schemes, aqueous sodium sulfate solutions of different concentrations and volumes (0.1–0.5 mol·L^−1^) were fed into the salt chamber (the compartment between the anion exchange membrane and cation exchange membrane) at different flow rates, and 500 mL deionized water (containing a few drops of the corresponding electrolyte solution) was fed into the acid chamber and the alkali chamber at different flow rates (200–600 mL·min^−1^) [23]. For circulation, 600 mL of 0.3 mol·L^−1^ aqueous Na_2_SO_4_ solution was added to the anode and cathode chambers which are connected together internally, and the flow rate was 200–600 mL·min^−1^. The BMED recovery experiment was carried out in electrostatic suppression mode, with the current density ranging from 10 to 50 mA·cm^−2^, and the voltage could be read from the DC power supply board. The salt chamber conductivity was measured using a portable conductivity meter (DDBJ-350, INESA Scientific Instrument Co. Ltd., Shanghai, China), and the experiment was stopped when the salt chamber conductivity was lower than 500 uS·cm^−1^.

### 2.3. Analysis and Calculations

The concentrations of H_2_SO_4_ and NaOH were determined using a digital titrator (Continuous RS, VITLAB, Muhltal, Germany), with phenolphthalein used as an indicator. The acid and alkali concentrations of the product were calculated using Equation (1) [25]:

(1) $$C_{t}=\frac{C_{c}\times V_{c}}{V_{r}}$$

where $C_{t}$ is the molar concentration of H_2_SO_4_/NaOH (mol·L^−1^) at time *t*, $C_{c}$ is the concentration of the NaOH/H_2_SO_4_ standard solution, and $V_{r}$ and $V_{c}$ are the volume of the acid compartment at time *t* and the volume of the titration solution, respectively. 

The current efficiency (*η*, %) of the BMED process was calculated using Equation (2) [26]:

(2) $$\eta =\frac{Z\left(C_{t}V_{t}-C_{0}V_{0}\right)F}{NIt}\times 100\%$$

where $C_{t}\;$and $C_{0}$ are the molar concentrations of sulfuric acid (mol·L^−1^) at times *t* and 0, respectively; $V_{t}$ and $V_{0}$ are the volumes of produced acid at time *t* and time 0, respectively; *Z* is the absolute ion valence (*Z* = 2 for sulfuric acid); *F* is the Faraday constant (96,485 C·mol^−1^); *I* (A) is the applied current; *N* is the repeating unit (*N* = 5) of the apparatus; and *t* (min) is the test time.

The energy consumption *E* (kW·h·kg^−1^) was calculated using Equation (3) [26]:

(3) $$E=\int_{0}^{t}\frac{UIdt}{C_{t}V_{t}M}\;$$

where *E* (kW·h·kg^−1^) is the energy consumption; *U* (V) is the voltage drop across the BMED apparatus; *I* (A) is the applied current; $C_{t}$ (mol·L^−1^) and $V_{t}$ (L) are the concentration and volume of feed concentration, respectively, at time *t*; and *M* is the molar mass of sodium sulfate (142.04 g·mol^−1^). 

## 3. Results and Discussion

### 3.1. Effect of Feed Concentration on BMED

The feed concentration is the key factor that can affect BMED performance [27]. For this reason, the influence of the feed concentration was investigated in the range of 0.1 to 0.5 mol·L^−1^. The current density was 30 mA·cm^−2^, and the flow rate of all solutions was 500 mL·min^−1^. 

**Figure 3:**
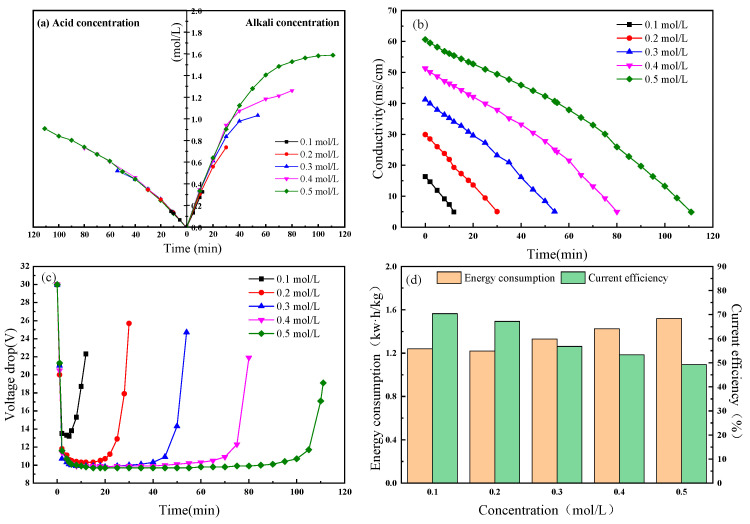
Effect of feed concentration on BMED performance: (**a**) acid concentration and alkali concentration, (**b**) electrical conductivity, (**c**) voltage drop across the apparatus, and (**d**) energy consumption and current efficiency.

Figure 3 shows that the C_H+_ in the acid compartment and C_OH−_ in the base compartment increased gradually as a function of time. It can be seen that the concentrations of acid and base increased as a function of time, indicating that H^+^ and OH^−^ were continuously produced by BPM and transported from the cation exchange layer (CEL) and anion exchange layer (AEL) to the acid and base compartments, respectively. Meanwhile, the SO_4_^2−^ ions were transported from the salt compartment to the acid compartment and combined with H^+^ ions to produce sulfuric acid, while the Na^+^ ions were transported from the salt compartment to the base compartment and combined with OH^−^ ions to produce sodium hydroxide. The greater the feed concentration, the higher the acid–base concentration obtained, which is in line with the theoretical situation. But we can see that as the salt concentration increases, the acid and base concentrations start to differ over time. This may be due to the following reasons. A fixed current density means a steady water splitting, resulting in a constant rate of proton formation. As the concentration of the salt chamber increases, high salt concentration leads to high osmotic pressure, which in this case increases the driving force for the diffusion of water molecules from the salt chamber to the alkali chamber, thus resulting in the enlargement of the volume of the alkali chamber. Figure 3b shows the change in salt chamber conductivity as a function of time. It can be seen that with the progress of the experiment, the conductivity of the salt chamber gradually decreased, and it could be reduced to 500 uS·cm^−1^ within the range of 0.1 to 0.5 mol·L^−1^, indicating that BMED has good conversion efficiency for sodium sulfate within the indicated concentration range. Figure 3c shows the voltage drop under different feed concentrations. It can be seen that the voltage drop decreased due to water splitting on the bipolar membrane at the beginning of the experiment. It then reached a plateau, and as the feed concentration increased, the voltage drop became smaller. In the later stage of the experiment, the concentration of the salt chamber dropped sharply, resulting in an increase in the resistance of the membrane stack and an increase in voltage drop. Figure 3d shows the current efficiency and energy consumption. It can be seen that the current efficiency decreased as feed concentration increased. According to Donnan equilibrium theory [28,29], the selectivity of an ion exchange membrane decreases with an increase in electrolyte concentration. As a result, more ions can be transported through the ion exchange membrane, resulting in a decrease in current efficiency at high salt concentrations. In addition, with the gradual increase in salt concentration, the osmotic pressure around the bipolar membrane also increases, and it becomes difficult for water molecules to migrate to the interfacial layer of the bipolar membrane, thus reducing the water supply required for water splitting. Theoretically, with increasing feed salt concentration, the resistance of the membrane stack decreases, and energy consumption decreases. However, when the feed salt concentration is greater than 0.3 mol·L^−1^, energy consumption starts to increase, which may be due to the large concentration gradient of sulfate ions in the acid chamber and salt chamber at the later stage of the experiment, leading to the diffusion of sulfate ions from the acid chamber to the salt chamber [30]. At the same time, the increased sulfate in the feed leads to an increase in osmotic pressure around the bipolar membrane, which inhibits water splitting in the bipolar interface of the bipolar membrane. Reduced water injection in a bipolar membrane interface increases energy consumption. Based on the comprehensive acid and base yield, it can be seen that when the salt concentration of the feed was about 0.2–0.3 mol·L^−1^, high current efficiency and low energy consumption could be achieved with high acid and base yield.

### 3.2. Effect of Current Density on BMED

**Figure 4:**
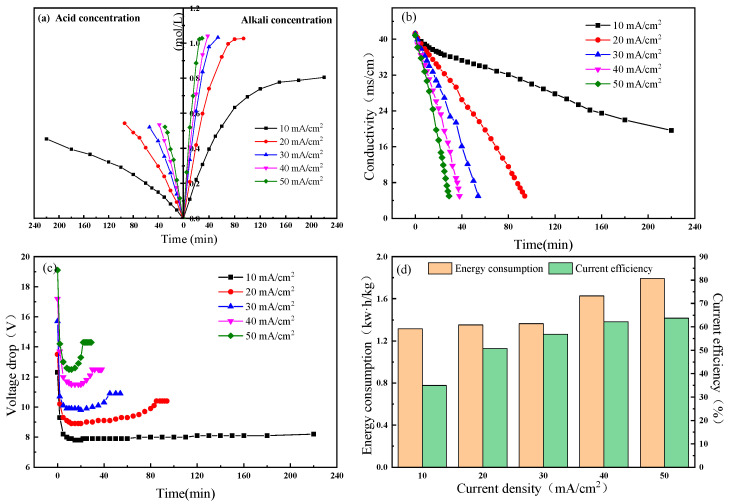
Effect of current density on BMED performance: (**a**) acid concentration and alkali concentration, (**b**) electrical conductivity, (**c**) voltage drop across the apparatus, and (**d**) energy consumption and current efficiency.

In the operation of BMED, current density is one of the most important factors that determine current efficiency, energy consumption, capital cost, and other operation performance indicators [21]. Current density in the range of 10 to 50 mA·cm^−2^, commonly used in BMED processes, was selected in this experiment. The concentration of sodium sulfate in the salt chamber was 0.3 mol·L^−1^. The flow rate of all circulating loops was 500 mL·min^−1^. Figure 4 shows the effect of current density on BMED operation. When the current density was 10 mA·cm^−2^, the experimental time was more than 240 min, and its desalting effect on sodium sulfate was very poor. Therefore, the experimental inflection point (after which desalting is considered unable to be carried out) was selected here for drawing processing. 

As shown in Figure 4a, the concentration of C_H+_ in the acid compartment and C_OH−_ in the base compartment increased with time, which indicates that sodium sulfate waste salt was successfully converted into sulfuric acid and sodium hydroxide, and the higher the current efficiency, the higher the concentration of generated acid and base. It can be seen from the diagram that when the current efficiency was 20–40 mA·cm^−2^, BMED was well able to convert sodium sulfate to acid and alkali. However, when the current density was 10 mA·cm^−2^, it can be seen from Figure 4b that the conductivity of the salt chamber decreased slowly. It took more than 240 minutes to reduce the conductivity of the salt chamber below 500 uS·cm^−1^, indicating that sodium sulfate decomposed when the current density was 10 mA·cm^−2^. The conversion efficiency was very low, and it was difficult to convert salt into acid and base. Figure 4c shows the variation curve of the voltage drop over time. Its basic trend is the same as that described above. Due to the low ion concentration in the initial acid and base chambers, the resistance of the membrane stack was larger. With the decomposition of the contents of the salt chamber by BMED, the concentration in the acid–base chamber increased gradually, and the resistance decreased and tended to a steady state [31]. In the late stage of the experiment, the salt chamber concentration decreased sharply, and the membrane stack resistance rose once again. In addition, the voltage drop increased with increasing current density, which is consistent with the theory [32]. Figure 4d shows the energy consumption and current efficiency with an increase in current density and a gradual increase in energy consumption; this fits most of the research results [33,34,35]. However, with increasing current density and current efficiency, high current density will, in theory, increase the water splitting, which increases with the amount of ion transport across the membrane; the current efficiency should then drop but, here, the opposite is observed. After ruling out a current density of 10 mA·cm^−2^ due to its desalination taking a long time with low current efficiency, the observations in the remaining four groups could be explained in three possible ways. Firstly, the concentration of the salt chamber is low, so the four groups of current density can effectively complete transformation; as a result, the influence of current density on the conversion rate is eliminated, and the difference in reaction time is small. The increase in the number of protons passing through the anion exchange membrane brought by high current density is not enough to reduce the current efficiency. In addition, due to the increase in current density, more H^+^ ions are generated near the bipolar membrane, which accelerates the reaction process and improves the current efficiency [36]. Lastly, in the case of consuming the same amount of charge, the acid chamber H^+^ ions increase with increasing current density, resulting in an increase in current efficiency [37]. Thus, there is a growing trend. In general, a current efficiency of 30 mA·cm^−2^ provided better processing performance.

### 3.3. Effect of Flow Rate on BMED

**Figure 5:**
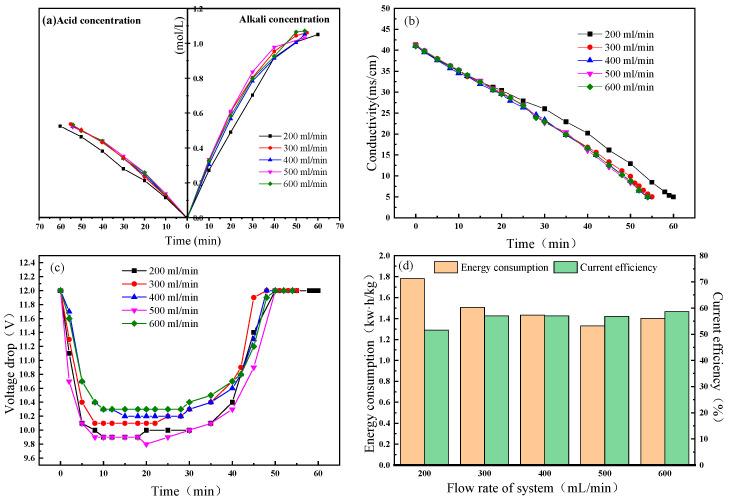
Effect of flow rate on BMED performance: (**a**) acid concentration and alkali concentration, (**b**) electrical conductivity, (**c**) voltage drop across the apparatus, and (**d**) energy consumption and current efficiency.

The influence of flow rate in the range of 200 to 600 mL·min^−1^ was studied. A current density of 30 mA·cm^−2^ was selected, and the sodium sulfate concentration in the salt chamber was 0.3 mol·L^−1^. Figure 5 shows the effect of flow rate on performance. The energy consumption for pump could be neglected compared with the energy applied for BEMD stack, so it was not considered in the experimental scale study.

Figure 5a shows the influence of flow rate on the acid and base yield, and it can be seen that the concentrations of acid and base increased as time passed. Figure 5b shows the change curve of salt chamber conductivity with time. It can be seen that with the passage of time, the conductivity values of the salt chambers with different flow rates were all reduced to 500 uS·cm^−1^. As the current density was the same, the salt concentration with a low flow rate needed a longer time. Figure 5c shows the effect of flow rate on the voltage drop of the BMED reactor. As the experiment progressed, the voltage drop showed a tendency to fall first, then to stabilize, and eventually rise. The initial descent was due to the introduction of acids and bases through the bipolar membrane. After about 7 minutes, the BMED reactor state began to stabilize, and the voltage drop also reached a stable state. It can be seen that as the flow rate increased, the voltage drop became larger and larger, and the time required for the experiment became shorter. Figure 5d shows the energy consumption and current efficiency at different flow rates. Energy consumption decreased with increasing flow rate mainly due to the short reaction time required by a high flow rate under the same current density. After reaching 500 mL·min^−1^, it started to rise again, which may have been because an increase in the feed liquid requires more energy for circulation. By contrast, the current efficiency was relatively stable, but the overall current efficiency still presented an upward trend. Since the number of H^+^ ions in the bipolar membrane was constant, the ratio of H^+^/Na^+^ at low flow rates was higher, and the current efficiency was lower [24]. Therefore, a flow rate of 500 mL·min^−1^ was more appropriate.

### 3.4. Effect of Volume Ratio on BMED

**Figure 6:**
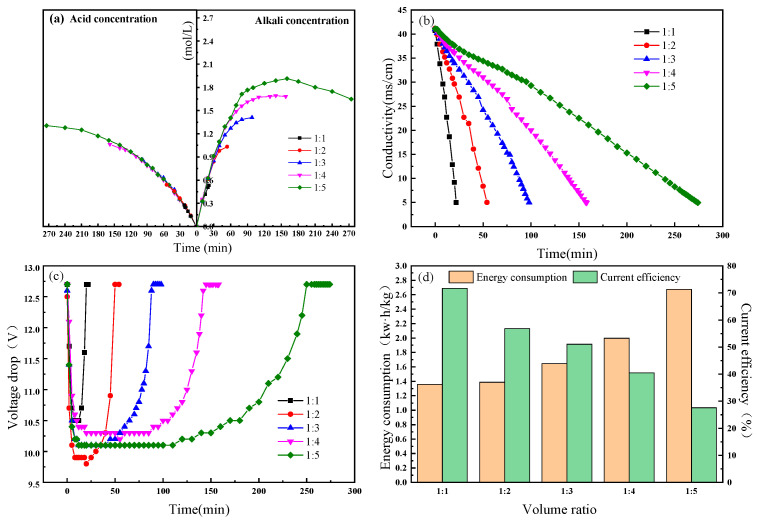
Effect of volume ratio on BMED performance: (**a**) acid concentration and alkali concentration, (**b**) electrical conductivity, (**c**) voltage drop across the apparatus, and (**d**) energy consumption and current efficiency.

Volume ratio, as one of the parameters of BMED operation, is also an important factor affecting performance [38]. Therefore, in this experiment, the volume ratio of the acid (alkali) chamber and the salt chamber was varied over 1:1, 1:2, 1:3, 1:4, and 1:5. We chose a current density of 30 mA·cm^−2^, and the sodium sulfate concentration in the salt chamber was 0.3 mol·L^−1^. The flow rate of all circulation loops was 500 mL·min^−1^. Figure 6 shows the effect of volume ratio on BMED performance. 

As shown in Figure 6a, the acid–base concentration increased with time. At the beginning of the experiment, the rates of acid and base increase in each experiment were not affected by the volume ratio. However, it is obvious that with increasing volume ratio, the final obtained concentration was higher. As the volume ratio gradually increased, the alkali chamber concentration was graded lower than the acid chamber concentration, and when the volume ratio reached 1:4, the concentration in the alkali chamber began to decrease. This was mainly due to the fact that when the volume of the salt chamber became larger, the experiment took longer, while the Na_2_SO4 content increased sharply with the increase of volumetric ratio. In this case, the osmotic pressure will also increase, which will increase the leakage of water, causing the alkali chamber to produce more water; this caused the volume of the alkali chamber to become larger, and the concentration then showed a downward trend [39]. This phenomenon can be attributed to the increased difference between the salt and acid chambers as the volume ratio increased. The number of sodium hydrate ions entering the alkali chamber was higher than the number of sulfate hydrate ions entering the acid chamber, and the amount of water carried by the hydrated ions also increased [40]. Figure 6b shows the change in conductivity of the salt chamber, and its change trend was basically the same as for acid and alkali concentration. Figure 6c shows the voltage drop versus time curve. The volume ratio had the same influence on the voltage drop as the flow rate. The initial voltage drop was mainly due to the fact that the initial acid–base chamber was pure water with very low conductivity. As the experiment progressed, acid and alkali were gradually produced, and the electrolyte concentration increased. Then, there was a plateau, and the voltage drop reached the lowest value. At this time, the average conductivity of the acid–base salt chamber was at a high level, and the BMED operation reached a steady state. In the later stage of the experiment, the voltage drop began to rise due to the greatly reduced conductivity of the salt chamber. When the volume ratio was 1:2, the voltage drop in the steady state was the lowest. The energy consumption and current efficiency are shown in Figure 6d. As the volume ratio increased, the current efficiency gradually decreased and dropped below 30% for a ratio of 1:5. This is mainly due to the increase in volume ratio—with the prolonging of reaction time, the concentration in the acid–base chamber increased, which led to an increase in ion back-diffusion and hydrolysis. The change in energy consumption was the opposite. When the volume ratio was 1:1, the energy consumption was 1.35 kWh/kg, and when the volume ratio was increased to 1:5, the energy consumption increased to 2.67 kWh/kg—an increase of more than 97%. On the basis of the high yield of acid and base, 1:2 was selected as the optimal volume ratio.

### 3.5. Performance Evaluation 

The above experiments demonstrate that the salt concentration, current density, flow rate, and volume ratio each have a significant influence on BMED performance. Through the BMED process, sodium sulfate wastes can be converted into sulfuric acid and sodium hydroxide, and the products can be used as raw materials in the upstream production process, thus achieving closed-loop production.

**Figure 7:**
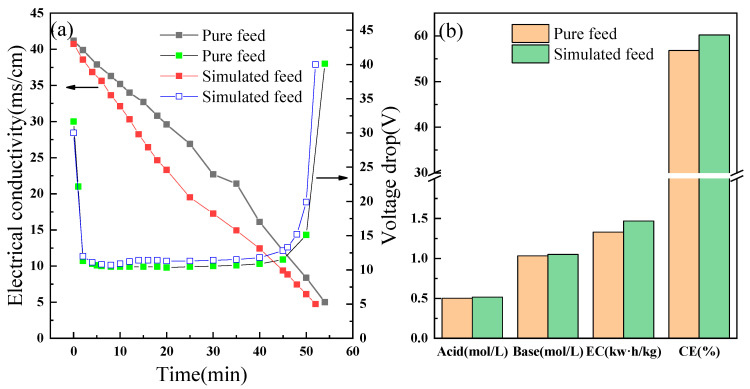
Comparison of BMED experiments using simulated feed and pure feed: (**a**) the experimental process and (**b**) the experimental results.

In order to further verify the effect of BMED on sodium sulfate waste salt treatment, a simulated waste salt solution was used for experiments. The treatment was performed under the abovementioned optimal experimental conditions. In addition, after each experiment, we used pure water circulation to clean the membrane stack for more than 30 min. As shown in Figure 7a, BMED efficiently converted the simulated salt solution into acid and alkali. The basic trend is consistent with the above. However, because the simulated salt solution contained other ions, the overall voltage drop was higher than that of the pure feed, and the reaction time was also shortened, which was mainly due to the presence of lithium ions and carbonate ions. It can be seen from Figure 7b that the final acid and base concentrations, current efficiency, and energy consumption were higher than the pure feed. When the solution contains lithium ions, the lithium ions and the sulfate ions may promote each other, which accelerates the reaction process and better transfers from the salt chamber to the corresponding compartment. At the same time, CO_3_^2−^ will combine with Na^+^ in the form of HCO_3_^+^, which leads to a relative increase in the migration amount and migration efficiency of lithium ions. The existence of CO_3_^2−^ has a beneficial effect on the experimental process to a certain extent. Table 3 lists the composition of the BMED products. The purity levels of sulfuric acid and sodium hydroxide reached 98.32% and 98.23%, respectively, as determined using an atomic absorption spectrograph (240 FS AA, Agilent Technologies, Inc., Santa Clara, CA, USA), which can be used in the production of lithium carbonate to achieve closed-loop production.

### 3.6. Economic Analysis 

We calculated the economic performance of the BMED process for treating sodium sulfate waste containing lithium carbonate, as shown in Table 4. The relevant data in Table 4 are based on previous calculation methods [41,42]. The processing capacity was calculated according to 8640 h per year with membrane stack cost being 1.5 times the membrane cost, and peripheral equipment cost being 1.5 times the membrane stack cost. The membrane was considered to have a service life of 3 years with an annual interest rate of 8% and maintenance cost of 10% of the total investment cost. Therefore, the cost of treating sodium sulfate with BMED was calculated as follows:

total fixed cost ($∙kg^−1^ Na_2_SO_4_) = total fixed cost ($·year^−1^)/process capacity (kg Na_2_SO_4_·year^−1^) 

total process cost ($∙kg^−1^ Na_2_SO_4_) = total fixed cost ($∙kg^−1^ Na_2_SO_4_)/total energy cost ($∙kg^−1^ Na_2_SO_4_)

It can be seen that under the premise of meeting processing standards, the cost of the process is USD 0.705 kg^−1^ Na_2_SO_4_. Taking into account the cost of originally disposing of sodium sulfate waste containing heavy metals and the benefits associated with the production of sulfuric acid and sodium hydroxide, the use of BMED for the treatment of sodium sulfate waste salt containing lithium carbonate is very beneficial.

## 4. Conclusions

Continuous and stable treatment of sodium sulfate wastes containing lithium carbonate can be achieved by using a process combining bipolar membranes and electrodialysis. BMED can convert sodium sulfate wastes into sulfuric acid and sodium hydroxide, both of which can be used as raw materials for upstream processes, realizing closed-loop production and presenting economic benefits through reduced costs. The effects of the feed salt concentration, current density, flow rate, and volume ratio on the performance of BMED were investigated. When the feed salt concentration was increased to 0.5 mol, the performance of BMED began to decline, which may have been due to the increase in osmotic pressure caused by the high concentration and the intensification of ion diffusion. To ensure acid–base yield, 0.2–0.3 mol·L^−^^1^ was selected as the best salt concentration for the feed. Regarding current density, high current density leads to high energy consumption. In order to maximize the economic benefits, we chose 30 mA·cm^−^^2^ as the optimal current density. The influence of the flow rate and volume ratio on BMED is also not to be underestimated. The best flow rate and volume ratio were 500 mL·min^−^^1^ and 1:2, respectively. Under the optimal technological conditions, the simulated wastewater treatment achieved the expected effect. The existence of lithium carbonate had a beneficial effect on the experimental process to a certain extent. The obtained sulfuric acid and sodium hydroxide products were determined by atomic absorption spectrometry, and the impurity content was low, with purity rates reaching 98.32% and 98.23%, respectively. The economic cost of the BMED process was calculated as USD 0.705 kg^−^^1^ Na_2_SO_4_. Therefore, BMED treatment of sodium sulfate waste salts containing lithium carbonate promotes the recycling production of lithium carbonate and has good economic and environmental benefits.

## Figures and Tables

**Table 1:** Chemical composition of simulated feed.

Components (mg·L^−1^)	Li^+^	Ca^+^	Mg^+^
Simulated feed	4500	3.28	0.20

**Table 2:** Properties of the membranes applied to the BMED experiments ^a^.

Membrane Characteristics	Neosepta AMX	Neosepta CMX	Neosepta BP-1
IEC (meq·g^−1^)	1.4–1.7	1.5–1.8	-
Thickness (μm)	120–180	220–260	200–350
Area resistance (Ω·cm^2^)	2.0–3.5	2.0–3.5	-
Voltage drop (V)	-	-	1.2–2.2
Current efficiency (%)	-	-	>98
Transport number (%)	91	98	>98

^a^ Data from the manufacturer’s instruction manual.

**Table 3:** Component analysis of BMED products.

	Product	Component (mg·g^−1^)				
		SO_4_^2−^	Na^+^	Li^+^	Ca^+^	Mg^+^
Feed	H_2_SO_4_ (98 wt. %)	- ^a^	12.99			
	NaOH (dry weight)	11.55	-			
Simulated feed	H_2_SO_4_ (98 wt. %)	-	17.06	0.07	-- ^b^	--
	NaOH (dry weight)	16.43	-	1.64	--	--

^a^ High content, did not detect the item; ^b^ low content, was not detected.

**Table 4:** Process cost analysis of bipolar membrane electrodialysis.

Parameters	BMED Process	Remarks
Feed volume (L)	1.0	
Feed salt concentration (mol·L^−1^)	0.3	
Current density (mA·m^−2^)	30	
Batch experiment time (min)	52	
Effective each membrane area (cm^2^)	189	
Energy consumption (kWh·kg^−1^ Na_2_SO_4_)	1.469	
Treatment capacity (kg Na_2_SO_4_·year^−1^)	424	1 year, 8640 h
Price of bipolar membrane ($·m^−2^)	800	
Price of mono membrane ($·m^−2^)	200	
Membrane lifetime and amortization of the peripheral equipment (year)	3	Based on the literature ^a^ and actual conditions
Electricity charge ($·kWh^−1^)	0.0825	Based on China’s electricity
Membrane cost ($)	128.52	
Apparatus cost ($)	192.78	
Peripheral equipment cost ($)	289.17	
Total investment cost ($)	481.95	
Amortization ($·year^−1^)	160.65	
Interest ($·year^−1^)	38.556	Interest rate, 8%
Maintenance ($·year^−1^)	48.195	10% of total investment cost
Total fixed cost ($·year^−1^)	247.401	
Total fixed cost ($∙kg^−1^ Na_2_SO_4_)	0.5835	
Energy cost ($·kg^−1^ Na_2_SO_4_)	0.1212	
Total process cost ($·kg^−1^ Na_2_SO_4_)	0.705	

^a^ H. Strathmann, and G.H. Koops, Process economics of electrodialytic water dissociation for the production of acid and base. in: A.J.B. Kemperman, (Ed.), Handbook on bipolar membrane technology, Enschede: Twente University Press, 2000, pp. 191–220.
